# Monthly Summary

**Published:** 1873-04

**Authors:** 


### MONTHLY SUMMARY.

---

*Death from Ether.*—Dr. Dunning, acting House Surgeon at Bellevue Hospital, N. Y., reports (*Medical Record*, Oct. 1, '72,) the case of J. S., aged 68, who was admitted into the hospital on August 2, 1876, suffering from a fracture of left femur, just below the trochanter. The patient was treated by a "Buck's retension" until Aug. 20, when it was decided to apply a plaster of Paris splint. In order to make sufficient retension, and at the same time prevent the pain of the operation, ether was ordered to be administered. The administration of the anæsthetic was slowly and carefully made, and after perhaps ten minutes, the patient was fully under its influence, and the operation begun. A few turns of the plaster had been made when the patient's breathing was observed to be rather frequent and gasping. The pulse was, however, full and regular. The thorax was compressed two or three times, and the patient's breathing again became normal. As these symptoms do not rarely occur during etherization, they excited no special alarm. The ether was, however, withheld from the patient four or five minutes, his respiration and pulse being normal. As he then began, however, to move about, and his muscles were becoming rigid, the ether cone was again applied. In a minute or two the assistant, who was giving the ether, observed the pupils to be dilating rapidly, and the breathing to cease. The heart was still beating. The ether cone was, of course, immediately removed, and artificial respiration was again used, and all the batteries obtainable in the hospital were put in operation, in an effort to resuscitate the patient. His muscles occasionally responded by a spasmodic movement, but no breathing again occurred. The efforts at resuscitation were continued about forty minutes, until all response to the action of the battery had ceased. Autopsy was made three hours after death, rigor-mortis was marked, blood was fluid, heart contained a little fluid blood, with a little atheroma at base of aortic valves. The lower lobe of right lung is œdematous, and its lower portion in a state of red hepatization. Ether was pure, and about ℥ij was used.—*Medical News and Library.*

---

*The Cure of Stammering.*—The mode of treatment followed by M. Chervin, of Lyons, in this affection, has lately been the subject of investigation by a commission appointed by the Department Council. The commissioners state that they find the system successful, rapid, and permanent in its effects; which opinion confirms those of earlier date, given by commissions appointed in France, Belgium, Spain, etc.

Eight patients, severely afflicted with stuttering, were submitted, under the observation of the commissioners, to the system of M. Chervin. They varied in age from ten to twenty-nine years, and none of them could speak without stammering to an extent most distressing to themselves and to those who heard and saw them. In some cases the act of speaking was accompanied with convulsive movements of the mouth and eyes; in others, with spasmodic respiratory movements. Some had stammered from their infancy; in others the defect had been caused by a shock to the nervous system. Ten days after they had been placed under M. Chervin's treatment they were seen by the commissioners, and each of them could then speak distinctly without stammering or hesitation; and on the 28th they were pronounced cured, speaking then with natural ease and rapidity.

The system is as follows. All mechanical contrivances are discarded, but he teaches the patient, by means of a large number of exercises, gradually to pronounce with distinctness vowels, consonants, syllables and sentences. He pays great attention to the act of respiration, which he seeks to regulate. He teaches his patients to take, at certain intervals, a slow but normal inspiration, which is succeeded by an even, continuous and loud expiration, during which pronunciatiou is effected. The course of treatment occupies twenty days, the time being divided into three periods. During the first the patient is restricted to complete silence, so that the old habit may be broken; during the second period the patient is taught to speak slowly and deliberately; and during the third period he acquires the practice of speaking fluently and without clipping the words. This method is stated to have succeeded in the most difficult cases, and the good results are said to be permanent; but this greatly depends on the patient, who must occasionally make use of the means which were first used to cure him.—*Medical Record.*

---

*Wear and Repair of the Brain.*—The notion that those who work only with their brains need less food than those who labor with their hands, has been the cause of untold mischief. Students and literary men have often been the victims of a slow starvation, from their ignorance of the fact that mental labor caused greater waste of tissue than muscular. According to a careful estimate, three hours hard study wears out the body more than a whole day of work on the anvil or the farm. "Without phosphorous, no thought," is a German saying; and the brain increases in proportion to the amount of labor which the organ is required to perform. This wear and tear of the brain are easily measured by careful examination of the salts in the liquid excretions. The importance of the brain as a working organ is shown by the amount of blood it receives, which is proportionately greater than that of any other part of the body. One-fifth of the blood goes to the brain, though its average weight is only one-fortieth of the weight of the body. This fact alone would be sufficient to prove that brain-workers need more food, and better, than mechanics and farm laborers.—*American Artisan.*

---

*Neuralgia.*—One of the best methods of treating neuralgia is that recommended by Frosseau.—[Trousseau.?] A tight top thimble is filled with cotton wool, and a few drops of strong aqua ammonia dropped on it. The open mouth of the thimble is then applied over the seat of pain for a minute or two, until the skin is blistered. The skin is then rubbed off, and upon the denuded surface a small quantity of morphia (gr. ¼) is applied. This affords almost instant relief. A second application of the morphia, if required, is to be preceded by first rubbing off the new formation that has sprung up over the former blistered surface. —*Medical Archives.*

---

*Pill for Neuralgia.*—

℞ Ext. Hyoscyamus,
Ext. Valerian,
White Oxide of Zinc, aa dr. j.—M.

Make the mixture into sixty pills. One pill may be taken every three hours. This is a favorite old prescription of the German and French physicians, and is mentioned in many of their works on therapeutics.—*Jour. of Materia Medica.*

---

*Ether versus Chloroform.*—If the force of statistics be of any value, ether, beyond question, is the agent which presents the most powerful claims and must obtain our confidence as the safest anæsthetic. The reports at the Medical Society of Virginia during this session are conclusive: by combining American and British statistics, the result is found to be as follows:

| Agents employed. | Deaths. | | Inhalations. | | | |
|---|---|---|---|---|---|---|
| Ether | 4 | to | 92 815, | or 1 | in | 223,204 |
| Chloroform | 53 | to | 155,260, | or 1 | in | 2,873 |
| Mixture of Chloroform and Ether | 2 | to | 11,176, | or 1 | in | 5,588 |
| Bichloride of Methylene | 2 | to | 10,000, | or 1 | in | 5,000 |

It is thus proved statistically that chloroform is eight times as dangerous as ether, twice as dangerous as a mixture of chloroform and ether, and, as far as experience goes, it is more dangerous than bichloride of methylene; in fact, chloroform is the most dangerous of all the anæsthetic agents in use. If statistics are of any value these should be startling and impressive ones. The report of the London Chloroform Committee states the result of careful investigation, that "ether is less dangerous than chloroform;" and that "with every care and the most exact dilution of the chloroform vapor, the state of insensibility may pass in a few moments into one of immediate death;" and the latest surgical work from America advocates ether as the most suitable anæsthetic.—*British Medical Journal.*

---

*Five Temporary Teeth at Forty.*—The *Canada Journal of Dental Science* reports the case of a lady who had five teeth removed at the age of forty, which, on examination, proved to be the temporary teeth, the permanent ones coming on after them.

---

*Bleached Tincture of Iodine.*—The *Pharmacist and Chemical Record* (July, 1872,) gives the following formula for the above preparation:

℞—Tincture of iodine..............................
Glycerine, pure.................................*aa* ℥i.
Sulphite of soda ...................................ʒi.

Rub the salt to powder in a small mortar, and add the glycerine gradually; then pour in the tincture of iodine and triturate gently until the solution is effected, and the mixture assumes an amber color.

It is claimed that the properties of iodine are increased by the addition of the sulphite of soda, and that the glycerine enhances its value and convenience for local application.—G.—*Boston Medical and Surgical Journal.*

